# CircRNA Arf3 suppresses glomerular mesangial cell proliferation and fibrosis in diabetic nephropathy via miR-107-3p/Tmbim6 axis

**DOI:** 10.1007/s10863-024-10027-w

**Published:** 2024-08-09

**Authors:** Linping Zhang, Gang Jin, Wei Zhang, Qiong Wang, Yan Liang, Qianlan Dong

**Affiliations:** Kidney Disease and Dialysis Center, Shaanxi Provincial People’s Hospital, NO.256 Youyi West Road, Beilin District, Xi’an, 710068 Shaanxi China

**Keywords:** Circ_Arf3, miR-107-3p, Tmbim6, Diabetic nephropathy, Mesangial cell, Fibrosis

## Abstract

Diabetic nephropathy (DN) is one of microvascular complication associated with diabetes. Circular RNAs (circRNAs) have been shown to be involved in DN pathogenesis. Hence, this work aimed to explore the role and mechanism of circ_Arf3 in DN. Mouse mesangial cells (MCs) cultured in high glucose (HG) condition were used for functional analysis. Cell proliferation was determined using 5-ethynyl-2’-deoxyuridine (EdU) and cell counting kit-8 assays. Western blotting was used to measure the levels of proliferation indicator PCNA and fibrosis-related proteins α-smooth muscle actin (α-SMA), collagen I (Col I), fibronectin (FN), and collagen IV (Col IV). The binding interaction between miR-107-3p and circ_Arf3 or Tmbim6 (transmembrane BAX inhibitor motif containing 6) was confirmed using dual-luciferase reporter and pull-down assays. Circ_Arf3 is a stable circRNA, and the expression of circ_Arf3 was decreased after HG treatment in MCs. Functionally, ectopic overexpression of circ_Arf3 protected against HG-induced proliferation and elevation of fibrosis-related proteins in MCs. Mechanistically, circ_Arf3 directly bound to miR-107-3p, and Tmbim6 was a target of miR-107-3p. Further rescue assay showed miR-107-3p reversed the protective action of circ_Arf3 on MCs function under HG condition. Moreover, inhibition of miR-107-3p suppressed HG-induced proliferation and fibrosis, which were attenuated by Tmbim6 knockdown in MCs. CircRNA Arf3 could suppress HG-evoked mesangial cell proliferation and fibrosis via miR-107-3p/Tmbim6 axis, indicating the potential involvement of this axis in DN progression.

**Supplementary Information:**

The online version contains supplementary material available at 10.1007/s10863-024-10027-w.

## Highlight

- Circ_Arf3 expression was decreased after HG treatment in MCs.
- Ectopic overexpression of circ_Arf3 suppresses MC proliferation and fibrosis under HG condition.
- Circ_Arf3 can indirectly regulate Tmbim6 expression via sponging miR-107-3p.
- The miR-107-3p/Tmbim6 axis mediates the protective effects of circ_Arf3 on MC function in the presence of HG.

**Supplementary Information:**

The online version contains supplementary material available at 10.1007/s10863-024-10027-w.

## Introduction

Diabetic nephropathy (DN) is a major microvascular complication of diabetes accounting for the main cause of end stage renal disease (Shrikanth and Nandini 2020). The typical hallmarks of DN are extracellular matrix (ECM) deposition and thickness in glomerular basement membrane and expansion of glomerular mesangium, finally resulting in tubulo-interstitial fibrosis and glomerulosclerosis (Gerrits et al. 2020; Tung et al. 2018; Yasuzawa et al. 2021). It has been revealed that diabetic condition or hyperglycaemia evokes profibrotic reactions, which ultimately cause glomerulosclerosis (Alicic et al. 2017; Mima 2022). Additionally, the loss of mesangial cell (MC) viability is considered as a significant pathological event in the diabetes-related renal function destruction (Tung et al. 2018; Ortiz-Muñoz et al. 2010). Therefore, further investigations on mesangial cell dysfunction under high-glucose (HG) conditions are thought to contribute to the prevention of DN.

Circular RNAs (circRNAs) are a new class of covalently closed endogenous non-coding RNAs produced by back splicing, and lack the 5’ end cap and 3’ end poly-(A) tails (Kristensen et al. 2019a, b). They are highly conserved, and are widely and stably expressed in a variety of bodily fluids and tissues, and play crucial roles in regulating cell biological processes (Han et al. 2018; Kristensen et al. 2019a, b). Therefore, circRNAs may be promising biomarkers used for disease prediction and treatment (Zuo et al. 2020). Importantly, recent studies have shown diabetes affect circulating expression of circRNAs, and dysregulated circRNAs has been proved to be associated with DN progression (Jin et al. 2020). For example, circRNA_010383 was found to suppress ECM accumulation in HG-treated MCs, and impeded renal fibrosis and proteinuria in DN mice by miR-135a/TRPC1 axis (Peng et al. 2020). Hu et al. showed that circRNA_15698 aggravated HG-evoked accumulation of ECM in mouse MCs through up-regulating TGF-β1 via miR-185 (Chen et al. 2019). Wang’s team suggested that circ_0037128 silencing could suppress the proliferation and fibrosis in MCs treated with HG via miR-17-3p/AKT3 axis. All these data hinted the implication of circRNA in DN pathogenesis (Wang et al. 2021). Circ_Arf3 (ID: mmu_circ_0000650) is produced by the back-splicing of the exon 2 to 5 of Arf3 gene on chr15:98570767–98,573,260. It was discovered to be decreased in the kidney tissue of DN mice (Mou et al. 2020). Here, we speculated that circ_Arf3 might prevent DN progression.

Hence, HG-induced MCs were used in this work to elucidate the biological function of circ_Arf3 on cell viability and fibrosis. Besides that, we explored whether the protective action of circ_Arf3 on MC function was mediated by microRNA (miRNA/miR)/mRNA axis based on the competitive endogenous RNA (ceRNA) hypothesis (Hansen et al. 2013; Salmena et al. 2011).

## Materials and methods

### Clinical samples

A total of 30 type 2 diabetes mellitus (DM) patients and 30 healthy donors were enrolled into this study. Serums were collected and immediately frozen in liquid nitrogen for further analysis. All subjects have been informed before study, and this study was approved by the Ethical Committee of Shaanxi Provincial People’s Hospital.

### Cell culture

The mouse MCs (Shanghai Academy of Life Science, Shanghai, China) were grown in DMEM (Gibco, Shanghai, China) plus 1% penicillin/streptomycin (Gibco) and 20% fetal bovine serum (FBS, Gibco) at 37℃ with 5% CO_2_.

MCs at 80% confluency were treated with 30 mmol/l D-glucose (Solarbio, Beijing, China) (high glucose group, HG) to imitate the growth environment of MCs in DN condition. Cells incubated with 5.5 mmol/l D-glucose plus 19.5 mmol/l mannitol were used as the control to imitate normal growth environment.

### Reverse transcription and quantitative real-time PCR (qRT-PCR)

Total RNA was extracted using TRIzol reagent (Takara, Dalian, China). The PrimeScript RT Reagent Kit (Takara) was applied for the synthesis of first-strand cDNA. The quantification of molecules was measured by qRT-PCR analysis with the TB Green Premix Ex Taq II (Takara). The relative fold changes were represented by a cycle threshold (Ct) value. GAPDH or U6 was as an internal control. The sequence of primers was listed in Table 1.

**Table 1:** Primers sequences used for qRT-PCR.

Name	Primers (5’-3’)	
circ_Arf3 (mmu_circ_0000650)	Forward	GCCTCGTTCACTCGCTCCC
	Reverse	GCTTTCCCCCTTTTTCTGC
Arf3	Forward	AGACACTACTTCCAGAACACCCA
	Reverse	CAGCCAGTCCAAGCCTTCATACA
mmu-miR-107-3p	Forward	AGAGCAGCATTGTACAGGGCTATC
	Reverse	CTCAACTGGTGTCGTGGA
GAPDH	Forward	GGCATCTTGGGCTACACTGAGGA
	Reverse	GGTGGGTGGTCCAGGGTTTCTTA
U6	Forward	CTCGCTTCGGCAGCACATATACT
	Reverse	ACGCTTCACGAATTTGCGTGTC
Tmbim6	Forward	AGCCATGAGACCGAGCAAAAGAG
	Reverse	CAAAGAAAAGGTTCCCCAGAGAG

### RNase R digestion and actinomycin D treatment

For RNase R digestion, approximately 3 µg of total RNA was treated with RNase R (3 U/µg, Geneseed, Guangzhou, China) or Mock for 30 min at 37℃. For Actinomycin D treatment, 5 µg/ml Actinomycin D (ActD; Geneseed) was used to incubate with MCs to block the *de novo* RNA synthesis. Finally, levels of circ_Arf3 and Arf3 mRNA were detected by qRT-PCR.

### Cell transfection

pCD5-ciR/circ_Arf3 overexpression plasmids and scrambled pCD5-ciR plasmids (Vector), miR-107-3p inhibitor (anti-miR-107-3p), mimic (miR-107-3p) and negative control oligos (anti-miR-NC or miR-NC), siRNA targeting Tmbim6 (si-Tmbim6) and the nontarget siRNA (si-NC) were constructed by Sangon (Shanghai, China). Lipofectamine 2000 (Invitrogen, Camarillo, CA, USA) was employed for cell transfection. After 48 h of transfection, MCs were stimulated with HG condition for subsequent functional experiments.

### 5-Ethynyl-2’-deoxyuridine (EdU) assay

MCs were seeded into a 96-well plate overnight and incubated with 50 µM EdU labeling solution (RiboBio) in growth medium for 2 h. After being settled with 4% paraformaldehyde for 30 min, cells were stained with Click-It reaction mixture for 30 min, followed by Hoechst 33,342 staining (100 µl). Finally, EdU positive cells were observed and calculated using a fluorescence microscope (Leica, Wetzlar, Germany).

### Cell counting kit-8 (CCK-8) assay

Single MCs (5 × 104 cells/ml) were grown in each well of a 96-well plate overnight, and then mixed with 10 µl CCK-8 solution in 100 µl DMEM medium for 2 h incubation. Lastly, the OD value was read at 450 nm using a microplate reader at indicated times to assess cell proliferation.

### Western blotting

Proteins were isolated by using RIPA lysis buffer (Beyotime, Shanghai, China) plus 1% proteinase inhibitor and quantified employing the BCA kit (KenGen Biotech, Nanjing, China). About 40 µg of proteins were loaded onto 8% SDS-PAGE for separating and then electrophoretically shift onto PVDF membranes (Millipore, Temecula, CA, USA). After being blocked with 5% skim milk powder at 37℃ for 1 h, membranes were probed with the specific primary antibodies against PCNA (1:2000, ab18197), α-smooth muscle actin (α-SMA) (1:1000, ab32575), Collagen I (Col I) (1:2000, ab138492), Fibronectin (FN) (1:1000, ab32419), Collagen IV (Col IV) (1:1000, ab6586) and GAPDH (1:5000, ab181602) (Abcam, Cambridge, MA, USA) all night at 4℃, and then incubated with HRP-conjugated secondary antibody (D110058, 1:4000, Sangon Biotech, Shanghai, China) at 37℃ for 2 h. Protein bands were quantified using an ECL reagent (Beyotime), and gray values were quantified by Image Lab software.

### Dual-luciferase reporter assay

The fragments of circ_Arf3 or Tmbim6 3’UTR comprising the binding sites of miR-107-3p or the mutant version without miR-107-3p binding sites were amplified and cloned into pGL3-Basic (Promega, Madison, WI, USA) to establish wild-type (WT) or mutated (MUT) circ_Arf3-WT/MUT or Tmbim6-WT/MUT luciferase reporter vector. Then 50 ng above recombinant plasmids, 10 ng pRL-TK Renilla and 50 nM miR-107-3p mimic or the control (miR-NC) were co-transfected into MCs using Lipofectamine 2000 for 48 h, and luciferase activities were detected using the Dual Luciferase Assay System (Promega).

### RNA pull-down assay

Biotin-labeled circ_Arf3 WT/MUT probe (Bio-circ_Arf3-WT/MUT) or biotin-labeled Tmbim6 WT/MUT probe (Bio-Tmbim6-WT/MUT) and nonsense control probe (Bio-NC) were synthesized by Geneseed Biotech (Shanghai, China). MCs were lysed by using lysis buffer, and cell lysates were then incubated with above biotinylated probes for 2 h. Upon incubation with M-280 Streptavidin magnetic beads (Invitrogen) for 1 h, the RNA complexes bound to the beads were purified, and the relative RNA level of miR-107-3p was detected with qRT-PCR.

### Animal experiments

This animal experiment was approved by the Ethical Committee of Shaanxi Provincial People’s Hospital. Healthy C57BL/6J mice (25–30 g, *n* = 12, male, 8-week-old) were purchased from Hunan Lake Jingda (Hunan, China), and maintained in standardized conditions. Diabetes mice were fed for 4 weeks with high-fat diet and then induced by injecting 55 mg/kg streptozotocin (STZ, Sigma-Aldrich, St. Louis, MO, USA) after 12 h of fasting every day for 5 consecutive days. Mice in the Sham group were fed with a standard rodent food and tap water for 4 weeks and then injected with an equal volume of citrate buffer. 72 h later, the fasting blood glucose (FBG) of mice was determined, and mice with FBG higher than 11.1 mmol/L were selected as successful diabetic mice model. At the end of the third week after injection, when the blood glucose of mice was ≥ 16.7mmol/L, and urine protein positive, the diabetic nephropathy mouse model was successfully established. Then all mice were anesthetized and kidneys tissues were harvested.

### Statistical analysis

The data from three repetitions were manifested as mean ± standard deviation (SD). The significance of differences between groups was analyzed using Student’s *t* test, Mann-Whitney or ANOVA with Tukey’s post-test. *p < 0.05 suggested statistically significant.

## Results

### Circ_Arf3 is a stable circRNA and is decreased in HG-induced MCs

**Fig. 1:**
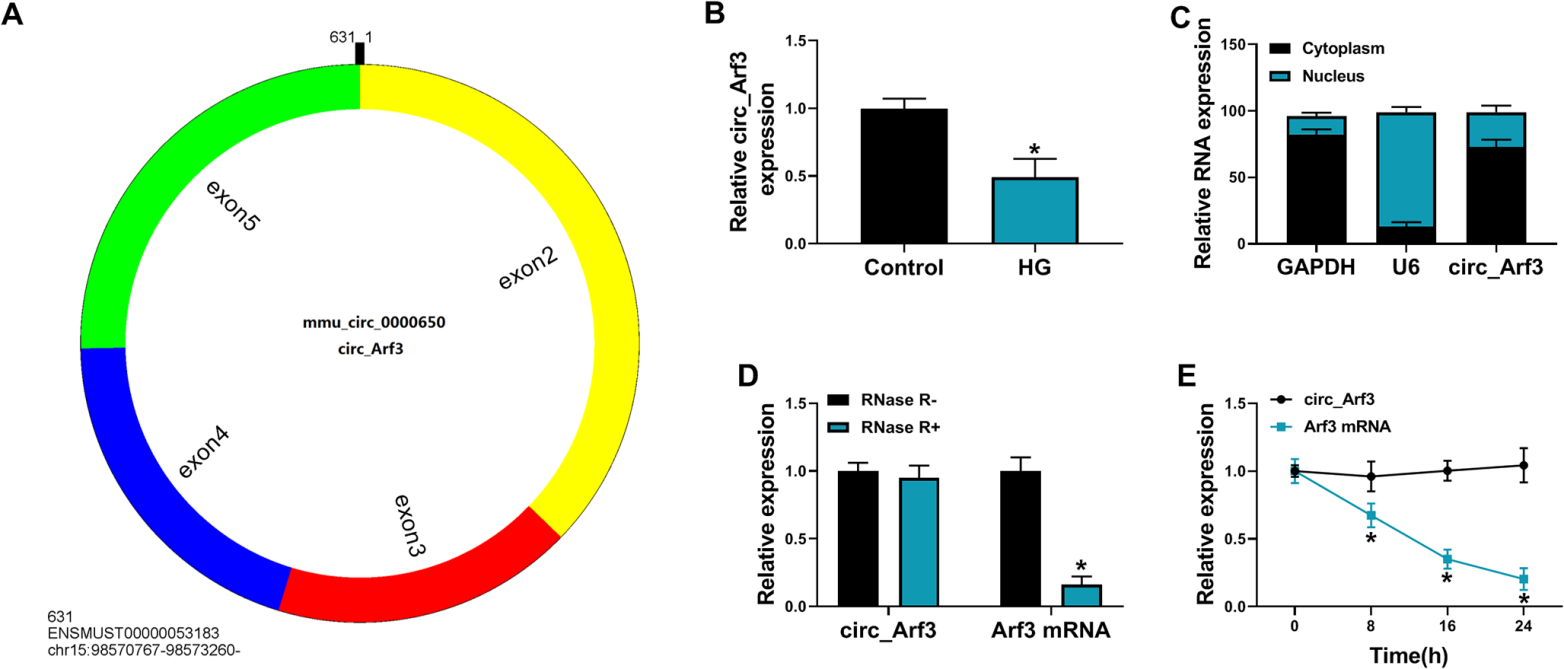
Circ_Arf3 is a stable circRNA and is decreased in HG-induced MCs. (**A**) The genomic locus and the back-spliced junction of circ_Arf3 were indicated. (**B**) qRT-PCR analysis of circ_Arf3 expression in MCs in HG condition or normal control condition. (**C**) The localization analysis of circ_Arf3 was conducted using nuclear-cytoplasmic fractionation assay. (**D**, **E**) RNase R and Actinomycin D treatment were used to evaluate the stability of circ_Arf3 in MCs. *p < 0.05

Circ_Arf3 is produced by the back-splicing of the exon 2 to 5 of Arf3 gene on chr15:98570767–98,573,260 with a length of 631 nt (Fig. 1A). We found that circ_Arf3 expression was decreased in the serum from human type 2 DM patients, and the kidneys tissues of DN mice (Fig. S1 and Fig. S2A). Then expression profile of circ_Arf3 in MCs was investigated by qRT-PCR, and results exhibited that circ_Arf3 expression was decreased after exposure with HG in MCs (Fig. 1B). Thereafter, nuclear-cytoplasmic fractionation assay showed that circ_Arf3 was mainly distributed in the cytoplasm of MCs (Fig. 1C). To investigate the stability of circ_Arf3 in MCs, RNase R and Actinomycin D were adopted. RNase R treatment could rapidly degrade linear Arf3 mRNA rather than circ_Arf3 in MCs (Fig. 1D). Besides that, the half-life of circ_Arf3 exceeded 24 h, while that of NNT mRNA was about 8 h in MCs (Fig. 1E), further validating the round structure of circ_Arf3. In all, circ_Arf3 is a stable circRNA and can function as a miRNA sponge, and dysregulated circ_Arf3 expression might be associated with MC dysfunction under HG condition.

### Circ_Arf3 overexpression suppresses the proliferation and fibrosis in MCs under HG condition

**Fig. 2:**
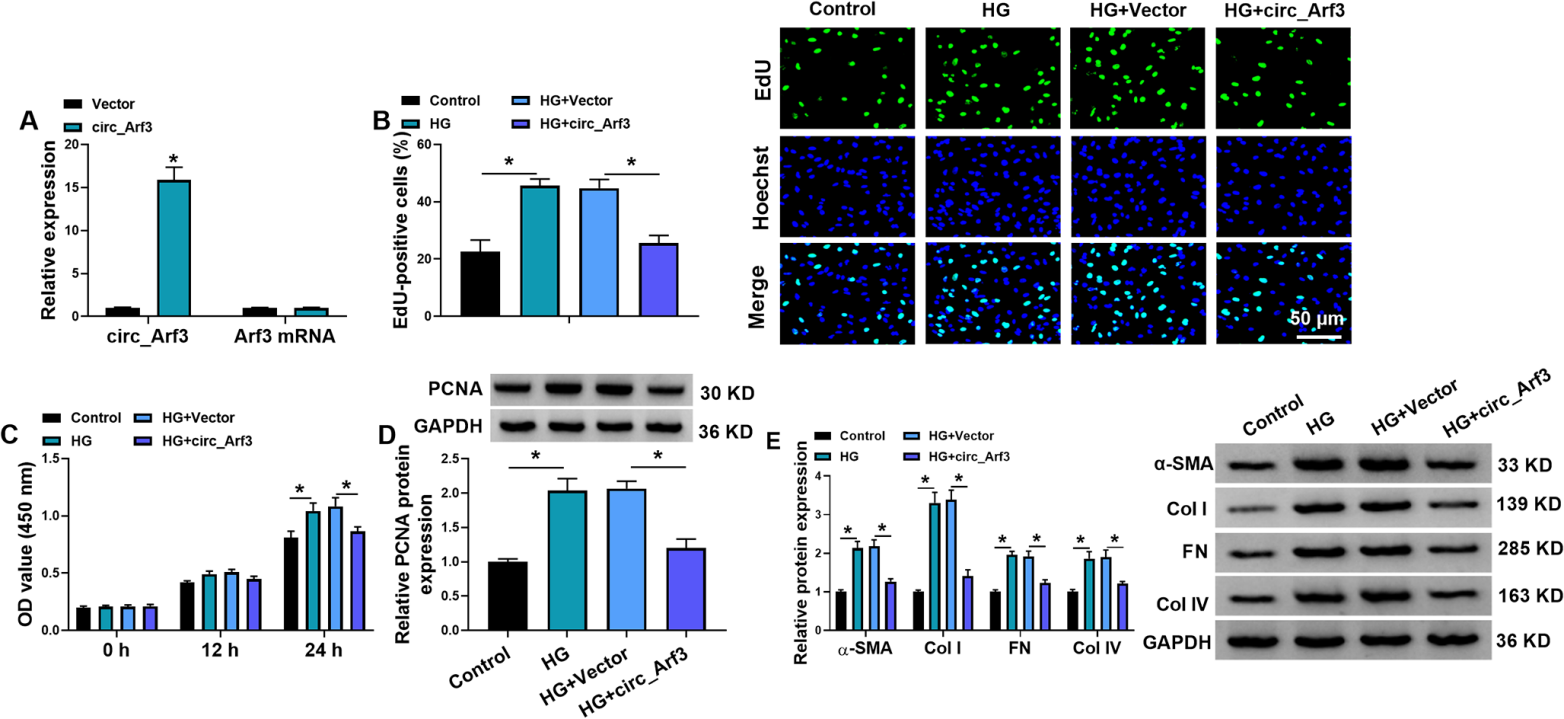
Circ_Arf3 overexpression suppresses the proliferation and fibrosis in MCs under HG condition. (**A**) The transfection efficiency of circ_Arf3 or Vector was validated using qRT-PCR in MCs. (**B**, **C**) The proliferation ability was measured in MCs transfected with circ_Arf3 or Vector under HG condition using EdU and CCK-8 assays. (**D**, **E**) The protein levels PCNA, α-SMA, FN, Col I, and Col IV in circ_Arf3 or Vector-transfected MCs under HG condition were detected using western blotting. *p < 0.05

To elucidate the functions of circ_Arf3 in DN, circ_Arf3 overexpression plasmids were constructed to investigate its functions. As expected, circ_Arf3 expression was significantly elevated after transfection in MCs (Fig. 2A). Functionally, circ_Arf3 overexpression suppressed HG-induced proliferation promotion in MCs, manifested by EdU and CCK-8 assays (Fig. 2B, C). Moreover, the protein level of PCNA, a proliferation indictor, was found to be increased in HG group, which was reduced by subsequent circ_Arf3 up-regulation (Fig. 2D). Besides that, the results of western blotting also showed that the expression levels of the well-known renal fibrosis-related factors, α-SMA, FN, Col I, and Col IV were decreased by circ_Arf3 overexpression in MCs under HG condition (Fig. 2E). Taken together, circ_Arf3 overexpression suppressed HG-induced MC proliferation and fibrosis.

### Circ_Arf3 acts as a sponge for miR-107-3p

**Fig. 3:**
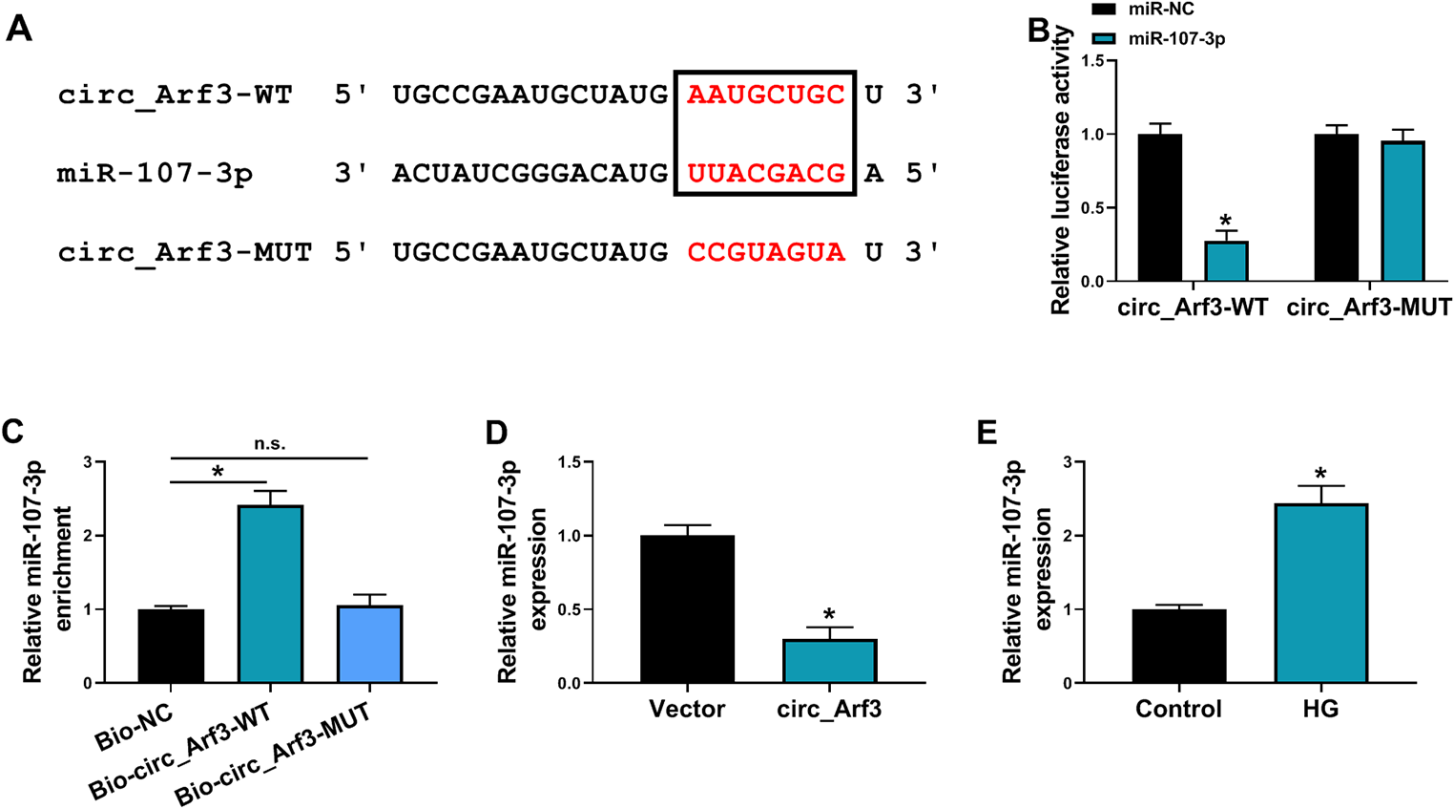
Circ_Arf3 acts as a sponge for miR-107-3p. (**A**) Schematic illustration of the binding site between circ_Arf3 and miR-107-3p. (**B**) Dual-luciferase reporter assay for the luciferase activity of the wild and mutated circ_Arf3 reporter vector after miR-107-3p overexpression in MCs. (**C**) qRT-PCR analysis of miR-107-3p level pulled down by biotin-labeled circ_Arf3 probe or control probe from lysates of MCs. (**D**) qRT-PCR analysis of miR-107-3p expression in MCs transfected with circ_Arf3 or Vector. (**E**) qRT-PCR analysis of miR-107-3p expression in MCs under HG condition or normal growth environment. *p < 0.05

Given that circ_Arf3 was mainly distributed in the cytoplasm of MCs, we first predicted the potential miRNAs of circ_Arf3 using starbase target prediction software. The data suggested that miR-107-3p had a putative target site on circ_Arf3 (Fig. 3A). Subsequently, the dual-luciferase reporter assay was executed, the results showed that miR-107-3p overexpression significantly reduced the luciferase activity of wild-type circ_Arf3 luciferase reporter but not the mutant one in MCs (Fig. 3B). Moreover, RNA pull-down assay suggested that miR-107-3p was markedly enriched by biotin-labeled circ_Arf3 probes compared with the control group in MCs (Fig. 3C), further verifying the binding between circ_Arf3 and miR-107-3p. Moreover, we also discovered that miR-107-3p expression was decreased by circ_Arf3 overexpression (Fig. 3D), but was increased in HG condition (Fig. 3E). Moreover, an increased expression of miR-107-3p in the kidneys tissues of DN mice was also observed (Fig. S2B). Thus, these results confirmed that circ_Arf3 directly targeted miR-107-3p and negatively regulated its expression.

### Circ_Arf3 suppresses MC proliferation and fibrosis under HG condition by repressing miR-107-3p

**Fig. 4:**
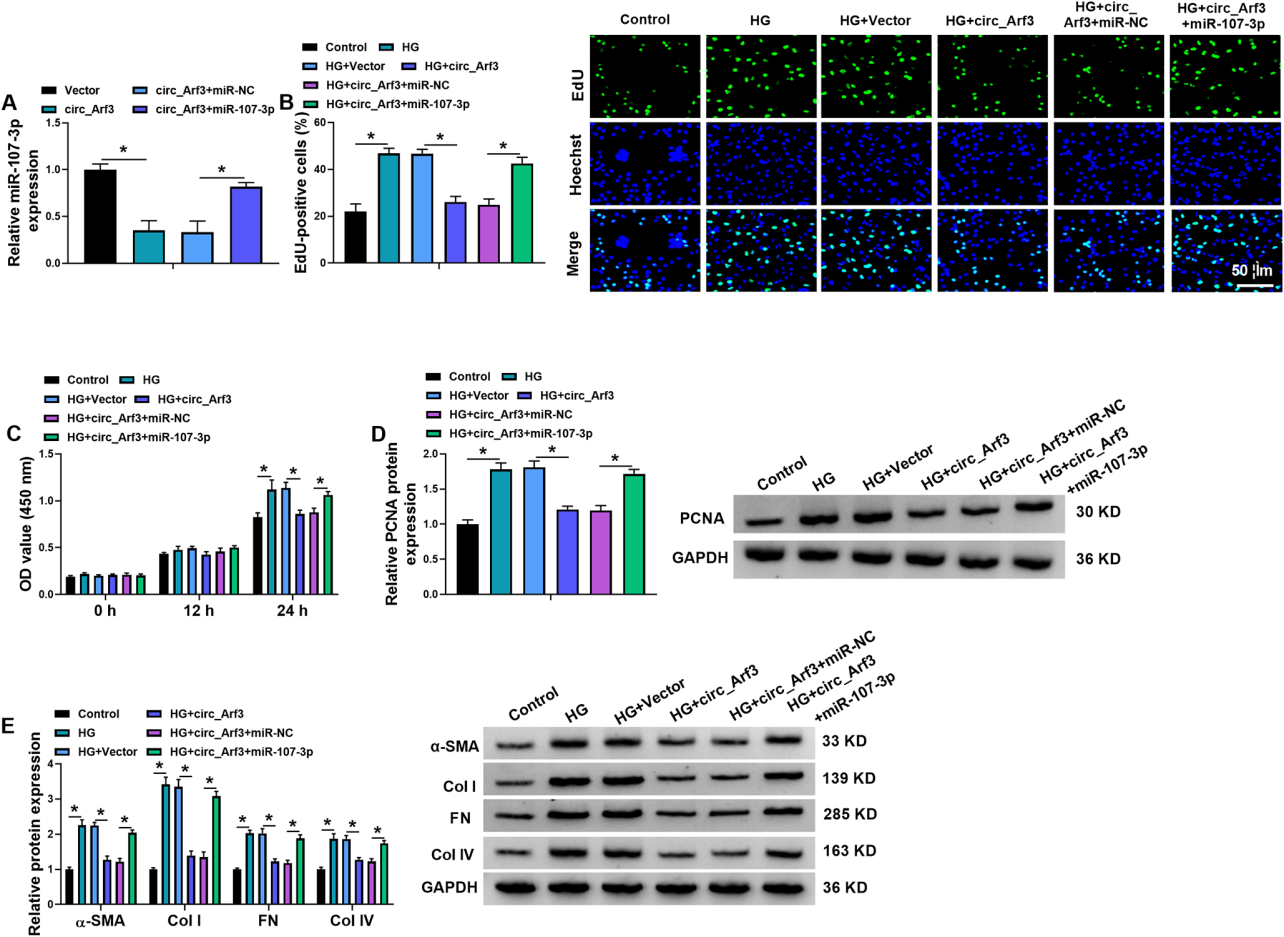
Circ_Arf3 suppresses MC proliferation and fibrosis under HG condition by repressing miR-107-3p. (**A**) MCs were co-transfected with Vector, circ_Arf3, circ_Arf3 + miR-NC, or circ_Arf3 + miR-107-3p, and then level of miR-107-3p was detected using qRT-PCR. (**B**-**E**) Transfected MCs were treated with HG. (**B**, **C**) The proliferation ability was measured in MCs using EdU and CCK-8 assays. (**D**, **E**) The protein levels PCNA, α-SMA, FN, Col I, and Col IV in MCs were detected using western blotting. *p < 0.05

To explore whether circ_Arf3 exerted its function by miR-107-3p, we conducted rescue assay. MCs were co-transfected with circ_Arf3 and/or miR-107-3p, the results of qRT-PCR showed that the introduction of miR-107-3p mimic significantly rescued circ_Arf3-induced decrease of miR-107-3p in MCs (Fig. 4A). Following HG treatment, it was proved that miR-107-3p mimic attenuated circ_Arf3-induced inhibition of cell proliferation in MCs under HG condition, which were accompanied with the decrease of PCNA in cells (Fig. 4B-D). Besides that, the decreases of α-SMA, FN, Col I, and Col IV protein levels mediated by circ_Arf3 overexpression in HG-induced MCs were abolished by miR-107-3p mimic (Fig. 4E). Thus, we demonstrated that circ_Arf3 might affect MC proliferation and fibrosis by miR-107-3p.

### Tmbim6 is a target of miR-107-3p, and circ_Arf3 can indirectly regulate Tmbim6 through sponging miR-107-3p

**Fig. 5:**
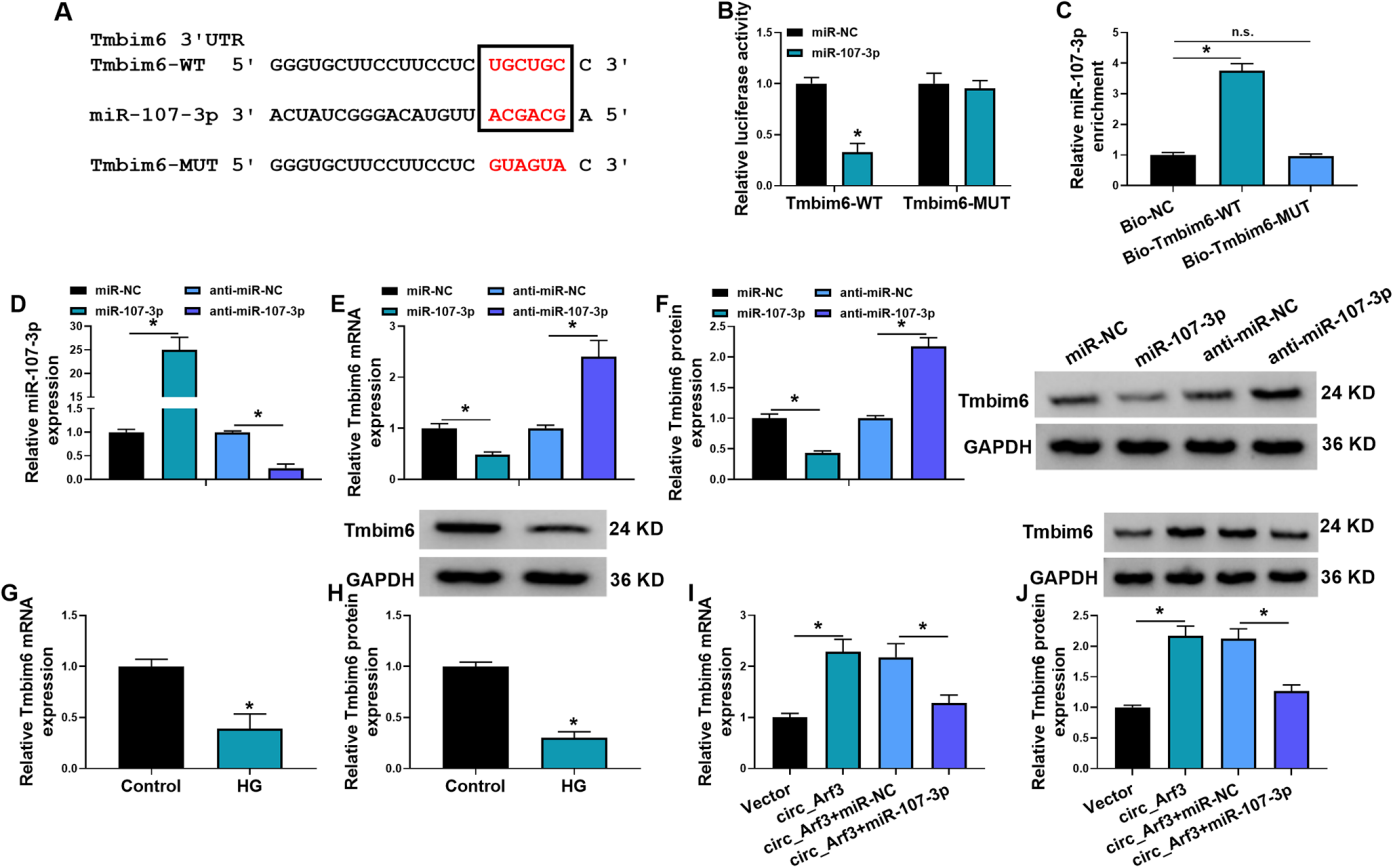
Tmbim6 is a target of miR-107-3p, and circ_Arf3 can indirectly regulate Tmbim6 through sponging miR-107-3p. (**A**) The complementary seed sequence between miR-107-3p and Tmbim6 3’UTR. (**B**) Dual-luciferase reporter assay for the luciferase activity of the wild and mutated Tmbim6 reporter vector after miR-107-3p overexpression in MCs. (**C**) qRT-PCR analysis of miR-107-3p level pulled down by biotin-labeled Tmbim6 probe or control probe from lysates of MCs. (**D**) The transfection efficiency of miR-107-3p mimic or the control was detected using qRT-PCR. (**E**, **F**) qRT-PCR and western blotting analysis of Tmbim6 level in MCs after miR-107-3p mimic or the control transfection. (**G**, **H**) qRT-PCR and western blotting analysis of Tmbim6 level in MCs treated with HG or not. (**I**, **J**) The effects of circ_Arf3/ miR-107-3p axis on Tmbim6 expression was validated using qRT-PCR and western blotting analysis. *p < 0.05

To explore the molecular mechanism underlying miR-107-3p, the potential targets of miR-107-3p were predicted using starbase database, and a complementary seed sequence was observed between miR-107-3p and Tmbim6 3’UTR (Fig. 5A). Thereafter, the results of dual-luciferase reporter assay showed that miR-107-3p overexpression declined the luciferase activity of wild-type Tmbim6 luciferase reporter, but failed to affect the luciferase activity of the mutated one in MCs (Fig. 5B). Furthermore, a specific enrichment of miR-107-3p in biotin-labeled Tmbim6 probe was observed (Fig. 5C). Next, the impact of miR-107-3p on Tmbim6 expression was investigated. The transfection efficiency of miR-107-3p mimic or inhibitor was firstly validated using qRT-PCR (Fig. 5D). Then we proved that miR-107-3p mimic reduced Tmbim6 expression, while miR-107-3p inhibitor up-regulated Tmbim6 expression in MCs (Fig. 5E, F). Moreover, Tmbim6 expression was lower in HG condition than those in normal growth condition in MCs (Fig. 5G, H). Besides that, we also observed a decrease of Tmbim6 expression in kidneys tissues of DN mice compared with the Sham group (Fig. S2C). All these data suggested the binding between Tmbim6 and miR-107-3p, and miR-107-3p could negatively regulate Tmbim6 expression in MCs.

Besides that, we also investigated the effects of circ_Arf3/miR-107-3p axis on Tmbim6 expression. It was found that circ_Arf3 overexpression led to an increase of Tmbim6 expression, which was reduced by miR-107-3p mimic (Fig. 5I, J), suggesting the circ_Arf3/miR-107-3p/Tmbim6 ceRNA network in MCs.

### Mir-107-3p inhibition restrains the proliferation and fibrosis in MCs under HG condition by Tmbim6

**Fig. 6:**
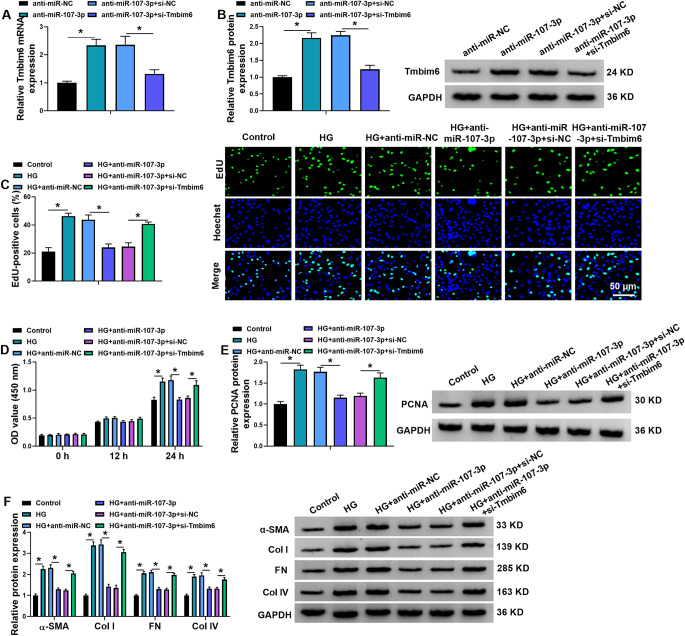
miR-107-3p inhibition restrains the proliferation and fibrosis in MCs under HG condition by Tmbim6. (**A**, **B**) MCs were co-transfected with anti-miR-107-3p and/or si-Tmbim6, and the levels of Tmbim6 were detected using qRT-PCR and western blotting analysis. (C-F) Transfected MCs were exposed to HG. (**C**, **D**) The proliferation of MCs was measured using EdU and CCK-8 assays. (**E**, **F**) The protein levels PCNA, α-SMA, FN, Col I, and Col IV in MCs were detected using western blotting. *p < 0.05

Next, the action of miR-107-3p/Tmbim6 axis in HG-induced MC dysfunction was investigated. MCs were co-transfected with anti-miR-107-3p and/or si-Tmbim6, then results manifested by Fig. 6A, B showed that the introduction of si-Tmbim6 reduced miR-107-3p down-regulation-induced elevation of Tmbim6 in MCs. Functionally, miR-107-3p silencing suppressed HG-evoked proliferation and PCNA elevation in MCs, which were reversed by Tmbim6 knockdown (Fig. 6C-E). Furthermore, levels of α-SMA, FN, Col I, and Col IV protein were reduced after miR-107-3p down-regulation in HG-induced MCs, while this phenomenon was counteracted by Tmbim6 knockdown (Fig. 6F). Collectively, miR-107-3p could regulate MC proliferation and fibrosis by Tmbim6 under HG condition.

## Discussion

In the last several decades, the incidence of DN has increased significantly, and it has become the leading cause of renal failure, while the current therapy of DN is limited to supportive treatment, such as blood pressure and hyperglycemic control (Fu et al. 2015). Thus, it is indispensable to better understand the pathogenesis of DN to develop effective therapeutic strategy for this disease. Previously, circRNAs have been proposed to benefit the pathological processes and the progression of renal fibrosis in DN (Liu et al. 2021; Tang et al. 2020). In our study, we identified that circ_Arf3 might be a significant signature circRNA in DN. It was found to be down-regulated in MCs under HG condition. Functionally, ectopic overexpression of circ_Arf3 in MCs reversed HG-induced proliferative activity, moreover, circ_Arf3 up-regulation reduced the accumulation of ECM-related proteins including α-SMA, FN, Col I, and Col IV in cells, thus preventing renal fibrosis. In all, circ_Arf3 up-regulation in mesangial cells may be an effective therapeutic approach for DN. However, although some interesting results were found in this study, the data presented are based on a limited number of cells in vitro, in vivo assay is essential to verify these conclusion using mice models with high or low circ_Arf3 expression in the future.

According to the ceRNA hypothesis (Hansen et al. 2013; Salmena et al. 2011), circRNA can function a sponge for miRNA, and liberate the degradation of downstream mRNA mediated by miRNA, moreover, the circRNA-miRNA-mRNA pathway in DN progression has been established (Mafi et al. 2021; Yao et al. 2020). Subsequently, whether circ_Arf3 exerted its effects by miRNA/mRNA axis was investigated. This study identified the circ_Arf3/miR-107-3p/Tmbim6 axis in MCs. Previous studies have showed the involvement of miRNAs in multiple pathological processes of DN, regulating pathways related to fibrosis, inflammatory response, or podocyte injury (Jiang et al. 2020; Guo et al. 2017; Zhao et al. 2019). Previous researches have showed that miR-107 can disturb glucose homeostasis and contribute to insulin resistance (Šimonienė et al. 2022; Yang et al. 2020). However, the action of miR-107-3p in DN remains unclear. In the current study, an increased miR-107-3p expression in MCs after HG treatment was observed. Functionally, miR-107-3p silencing attenuated HG-induced proliferation and fibrosis. Moreover, miR-107-3p could reverse the protective effects circ_Arf3 on MCs under HG condition. Tmbim6 is a highly conserved multi-transmembrane protein among species that has been identified to be involved in regulating the suppression of BAX-mediated cell death (Xu and Reed 1998). The deficiency of Tmbim6 suppressed glucose metabolism and could lead to obesity (Philippaert et al. 2020). Besides that, Wu et al. showed that miR-27a-3p down-regulation could suppress DN progression by regulating mitochondrial dysfunction, renal fibrosis, and endoplasmic reticulum stress through Tmbim6 (Wu et al. 2021). Consistent with the previous research, Tmbim6 was decreased by HG treatment in MCs, importantly, rescue experiments confirmed that Tmbim6 counteracted the suppressive functions of miR-107-3p on MC proliferation and fibrosis in the presence of HG.

**Fig. 7:**
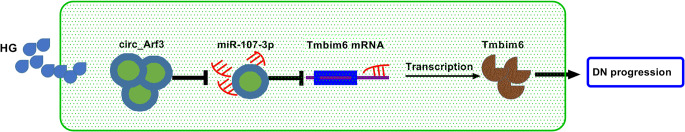
The schematic diagram illustrates how circ_Arf3 regulate DN progression. Circ_Arf3 up-regulates Tmbim6 through sequestering miR-107-3p to suppress HG-induced proliferation and fibrosis, and then hindering DN progression

In all, our study for the first time evidenced that circ_Arf3 could restrain mesangial cell proliferation and fibrosis via miR-107-3p/Tmbim6 axis under HG condition (Fig. 7), which might provide a new approach for DN prevention.

## Electronic supplementary material

Below is the link to the electronic supplementary material.

Supplementary Material 1

## Data Availability

The analyzed data sets generated during the present study are available from the corresponding author on reasonable request.

## Declarations

### Animal studies

Animal Studies: Animal studies were performed in compliance with the ARRIVE guidelines and the Basel Declaration. All animals received humane care according to the National Institutes of Health (USA) guidelines.
