# Supplementary material for: CircRNA Arf3 suppresses glomerular mesangial cell proliferation and fibrosis in diabetic nephropathy via miR-107-3p/Tmbim6 axis
Source: J Bioenerg Biomembr. 2024 Aug 9;56(5):543–52. doi: 10.1007/s10863-024-10027-w (PMC11455692; doi:10.1007/s10863-024-10027-w)
Supplement: Supplementary file 1 — Supplementary Material 1 [file 10863_2024_10027_MOESM1_ESM.pdf]

Marker-1 (M1)

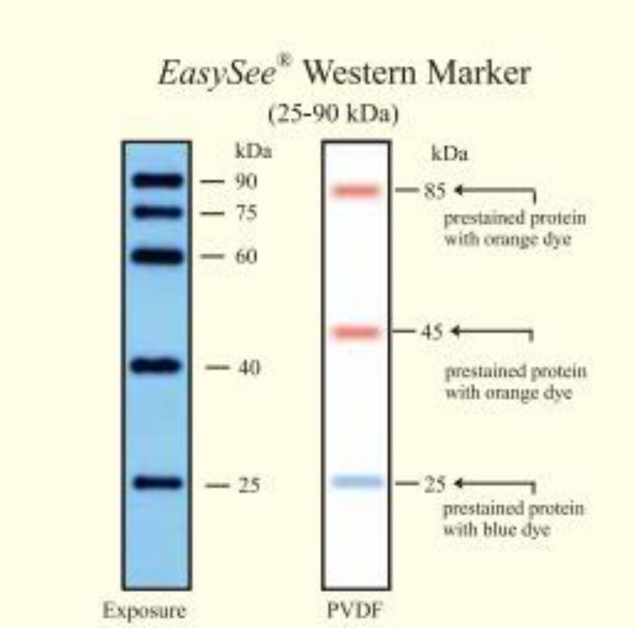

Marker-2 (M2)

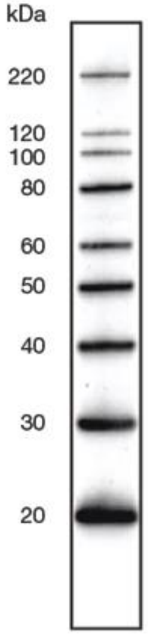

Fig2 D

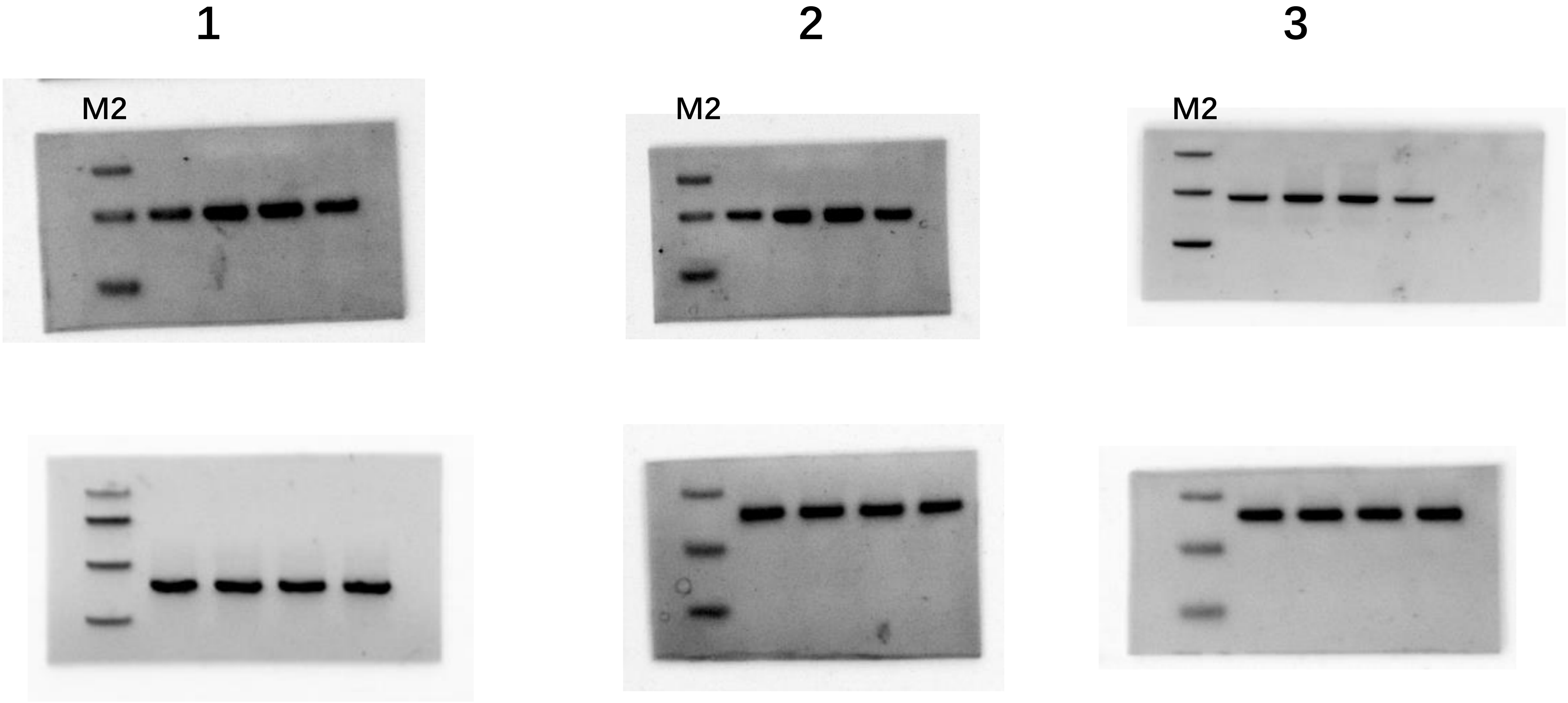

PCNA
(30 KD)

GAPDH
(36 KD)

Fig2 E

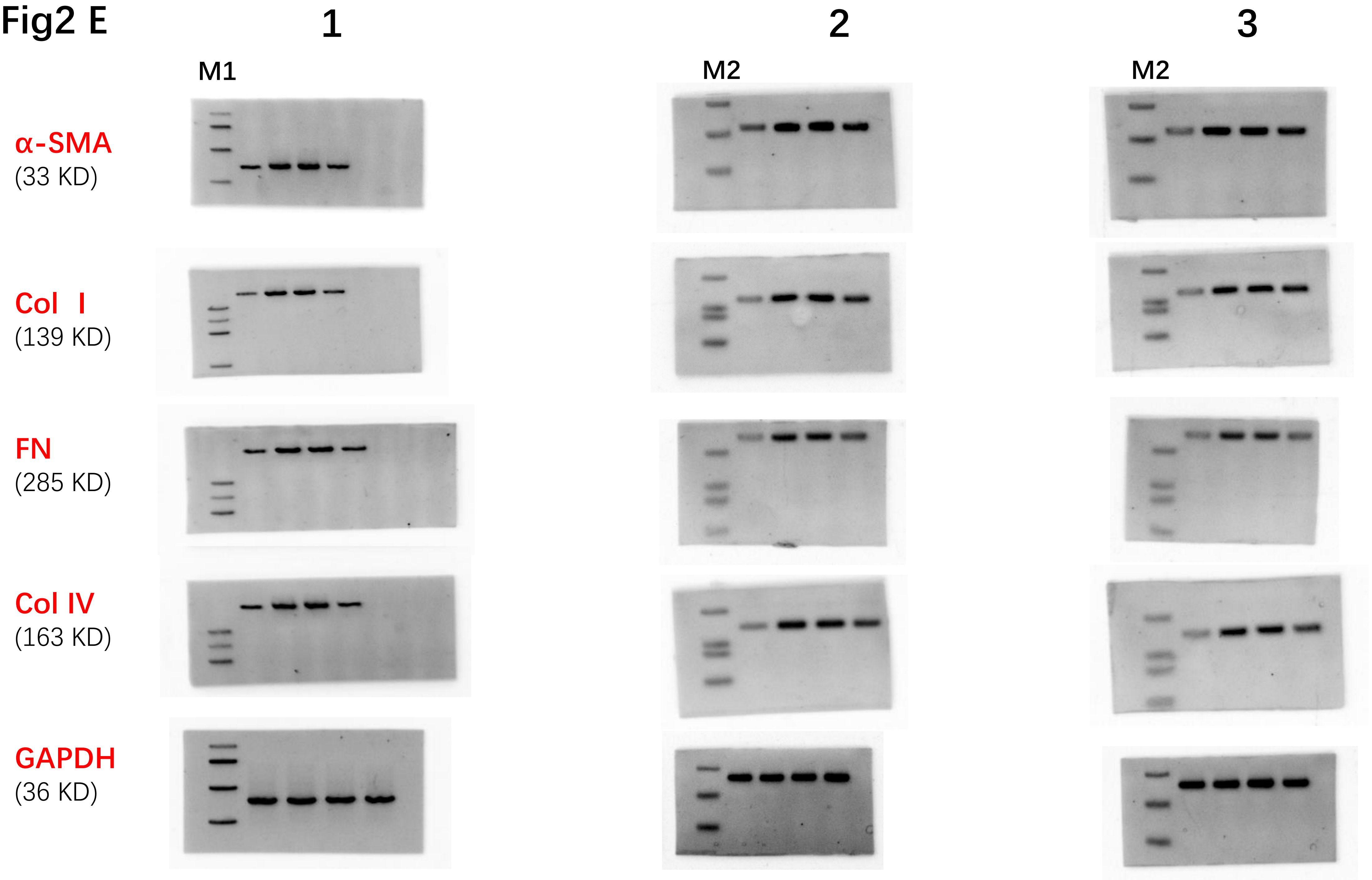
1
2
3
M1
M2
M2
α-SMA
(33 KD)
Col I
(139 KD)
FN
(285 KD)
Col IV
(163 KD)
GAPDH
(36 KD)

Fig4 D

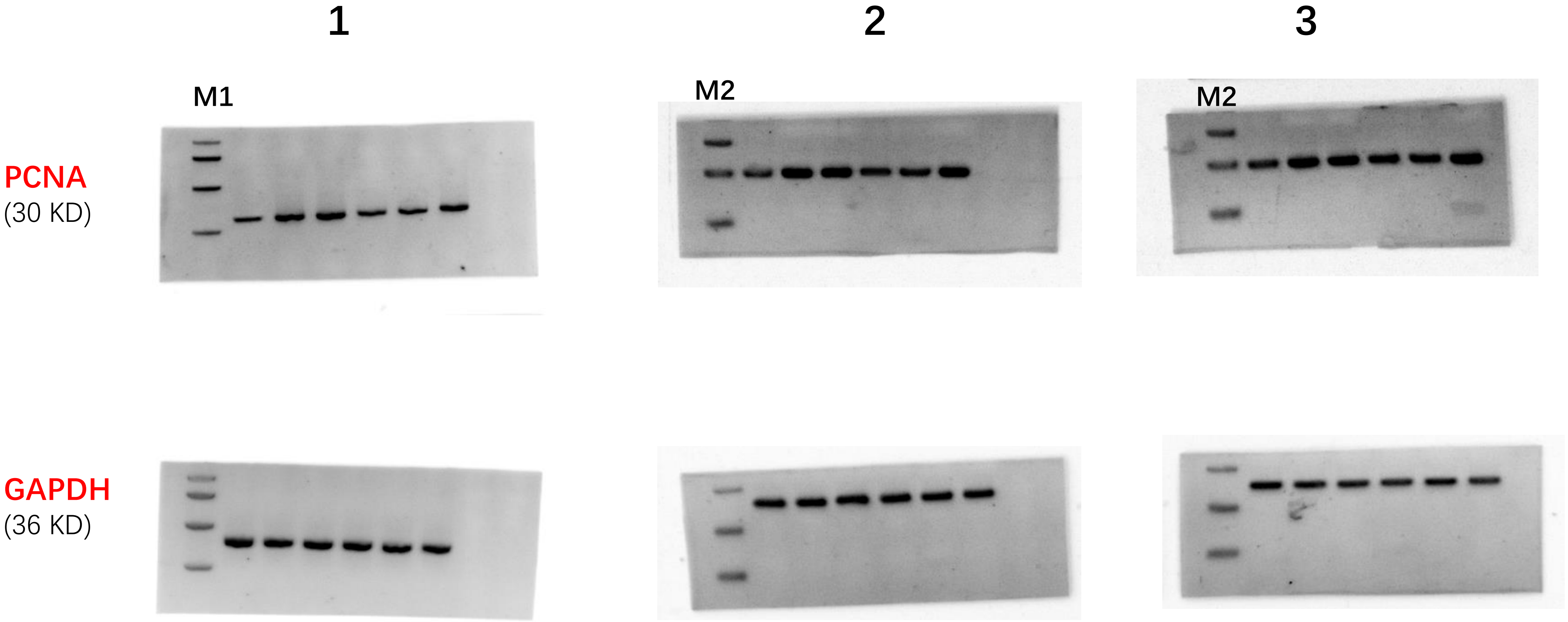

Fig4 E

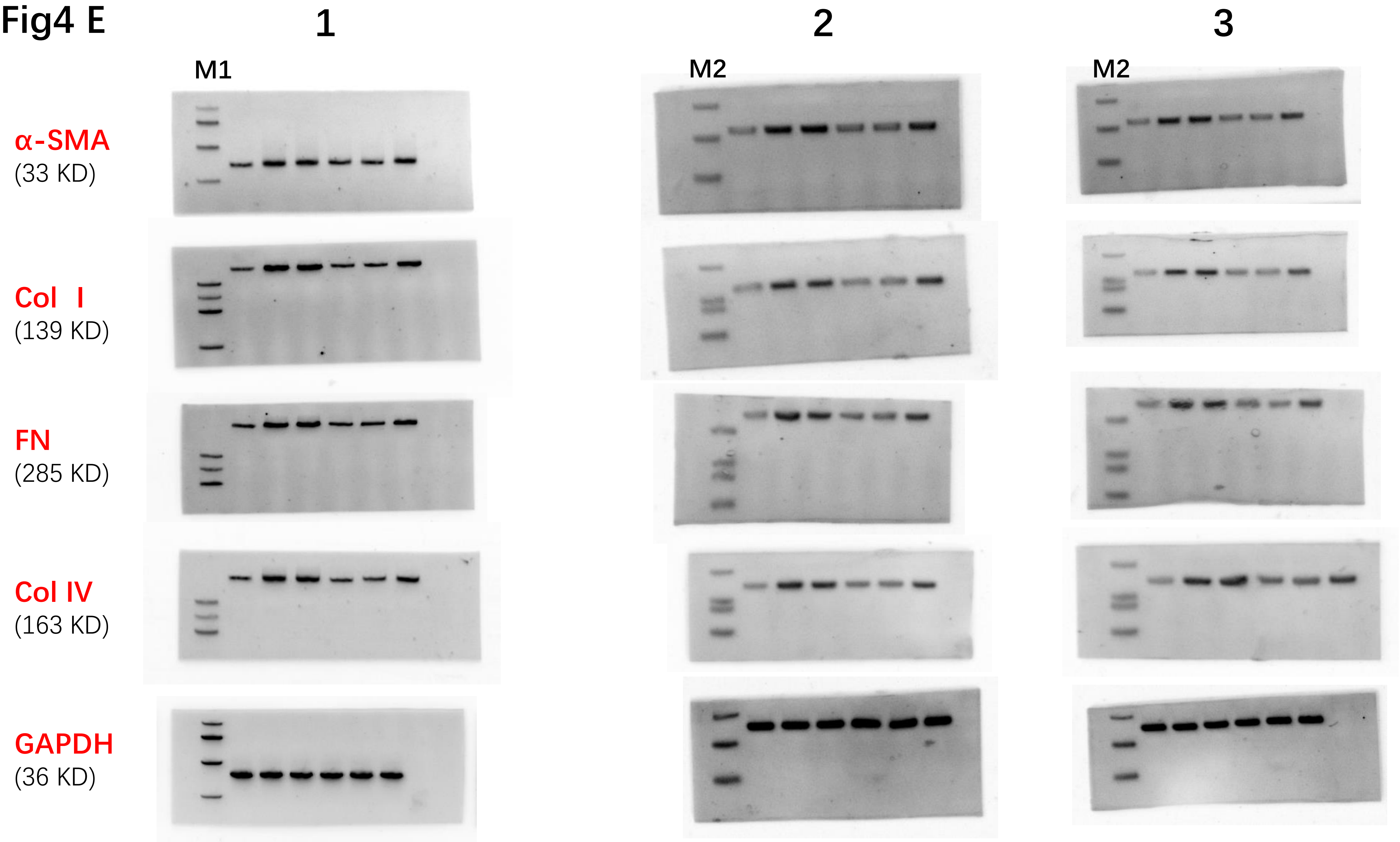
1
2
3
M1
M2
M2
α-SMA
(33 KD)
Col Ⅰ
(139 KD)
FN
(285 KD)
Col IV
(163 KD)
GAPDH
(36 KD)

Fig5 F

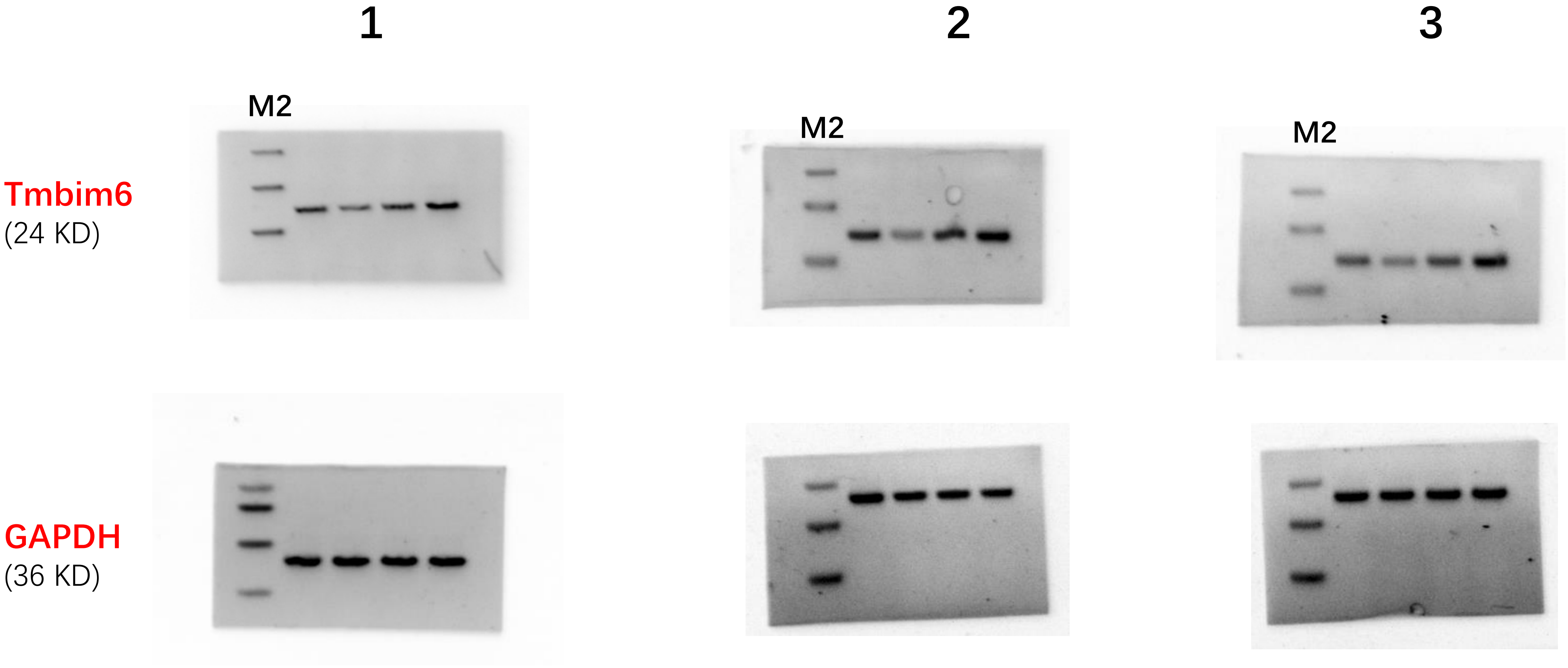

Fig5 H

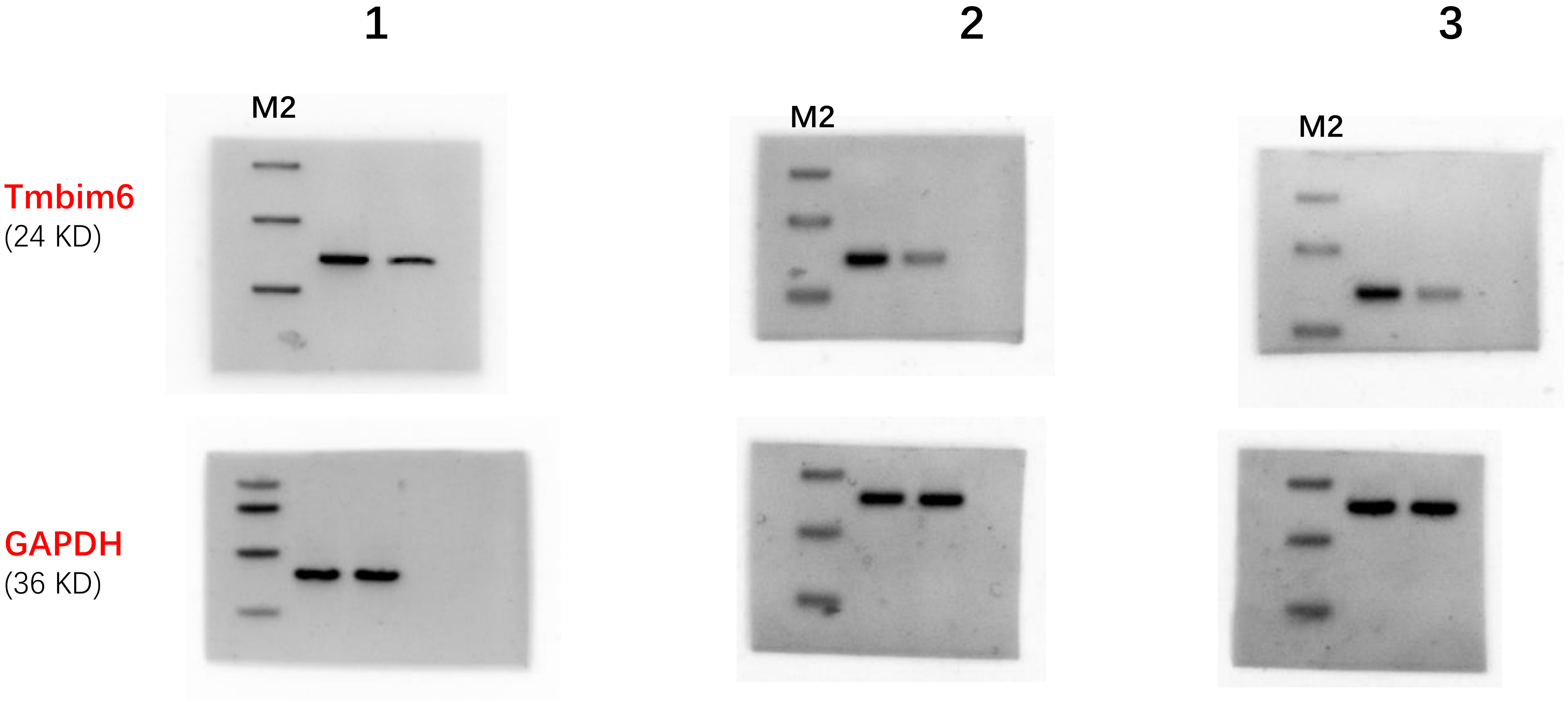

Fig5 I

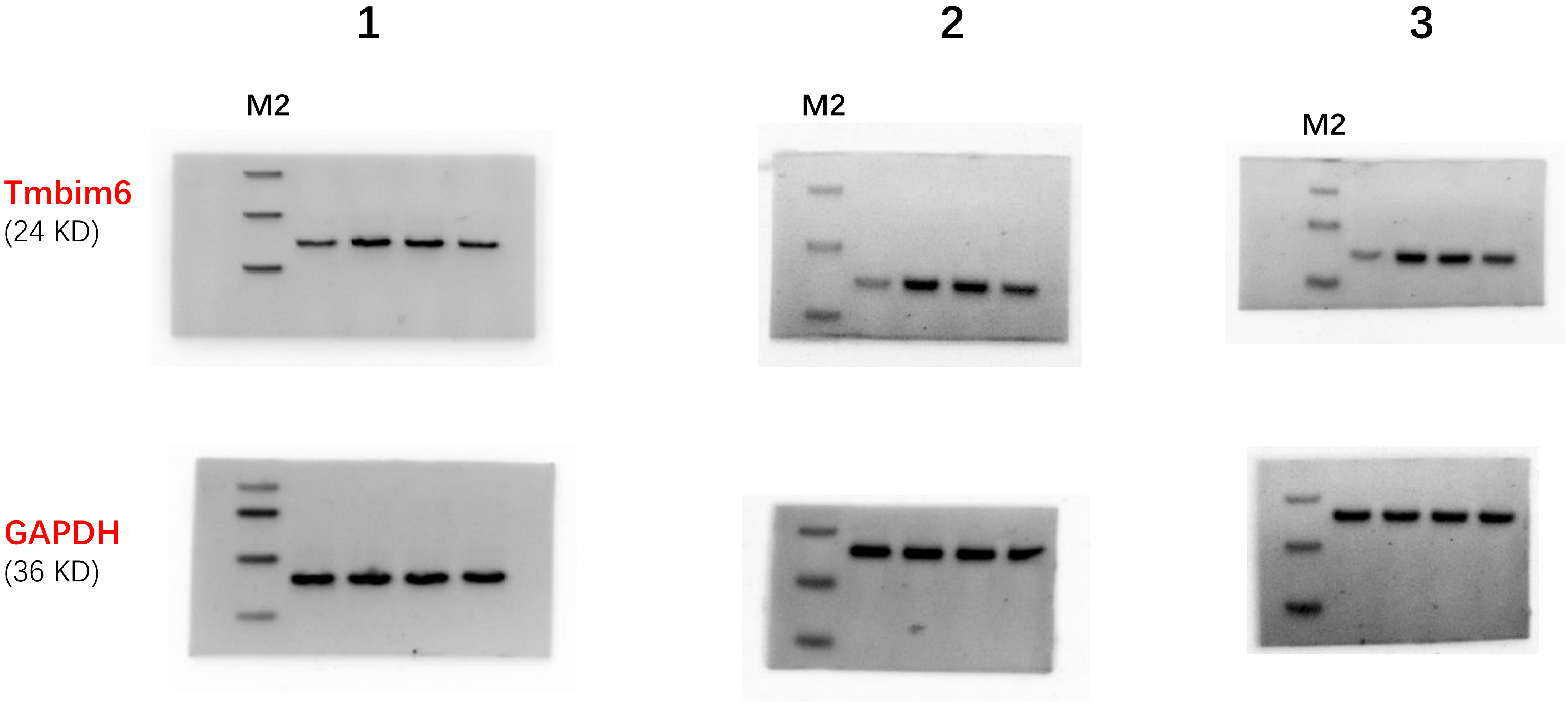

Fig6 B

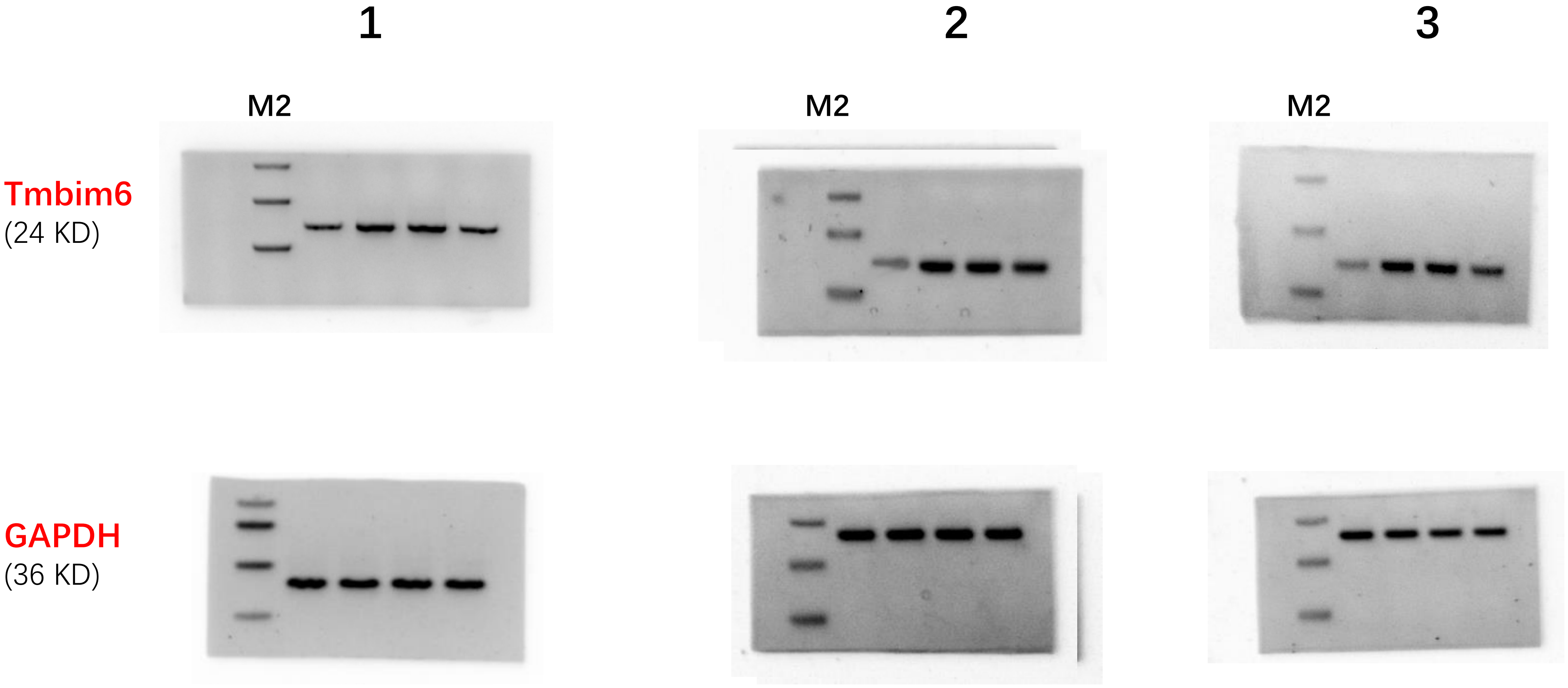

Fig6 E

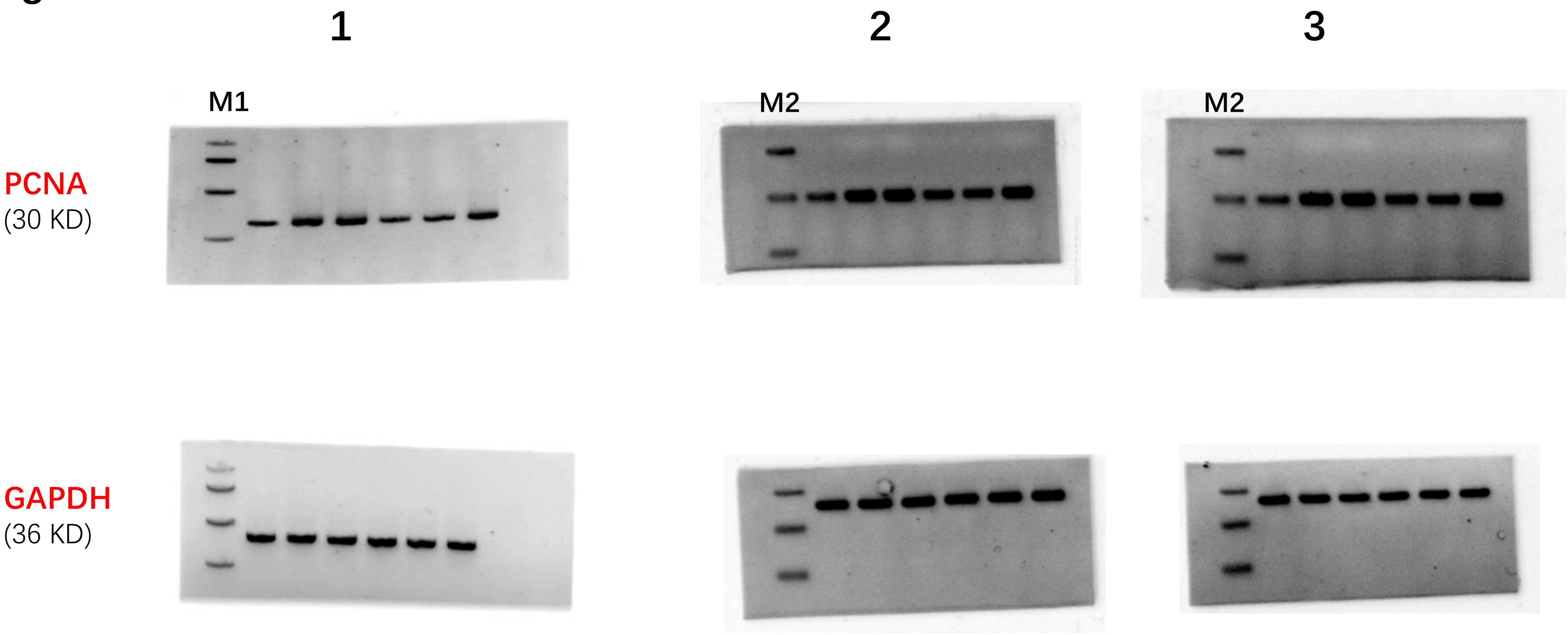

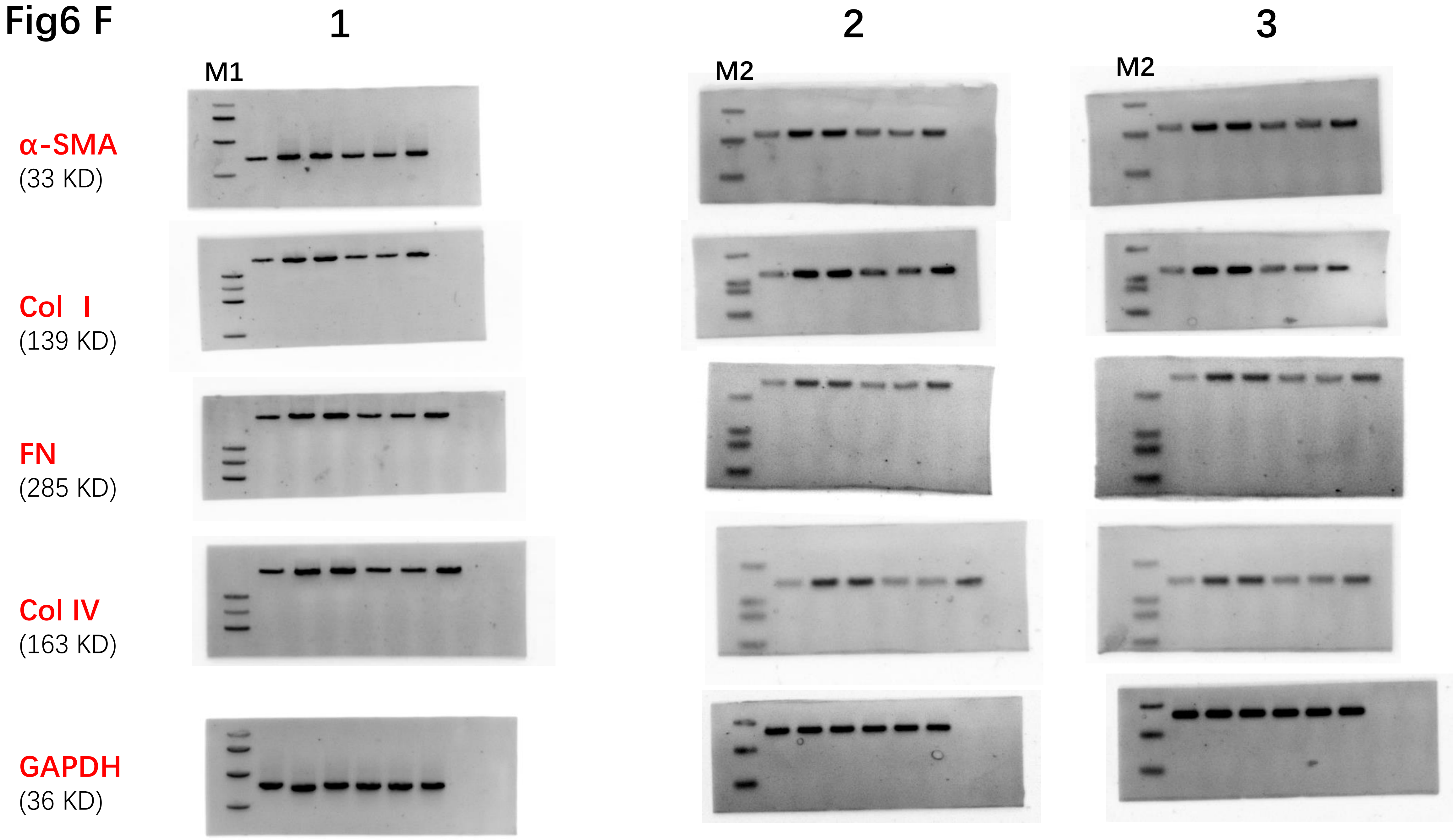
Fig6 F
1
2
3
M1
M2
M2
α-SMA
(33 KD)
Col I
(139 KD)
FN
(285 KD)
Col IV
(163 KD)
GAPDH
(36 KD)
